# Isolation of multidrug resistance bacteria from the patients with wound infection and their antibiotics susceptibility patterns: A cross-sectional study

**DOI:** 10.1016/j.amsu.2022.104895

**Published:** 2022-11-14

**Authors:** Fahim Alam Nobel, Saiful Islam, Golap Babu, Sharmin Akter, Ruksana Akter Jebin, Titash Chandra Sarker, Ashekul Islam, Mohammod Johirul Islam

**Affiliations:** a Department of Biochemistry and Molecular Biology, Mawlana Bhashani Science and Technology University, Santosh, Tangail, 1902, Bangladesh; b Department of Biochemistry and Molecular Biology, Jahangirnagar University, Savar, Dhaka, 1342, Bangladesh

**Keywords:** Antibiotic resistance, Antibiotic susceptibility test, Multi-drug resistance, Antimicrobial resistance, Gram-staining

## Abstract

**Introduction:**

Antimicrobial resistance has become one of the most severe public problems in both developed and developing countries like Bangladesh. In this study, several multi-drug resistant bacteria were isolated from the wound infections and demonstrated their antibiotic susceptibility pattern in Bangladeshi patients.

**Methods:**

A total of 699 bacterial isolates were collected from wound swabs and each isolate was identified using gram staining, biochemical assays, antibiotic susceptibility tests with the disk diffusion method, and colony morphology. Samples were taken from January 2018 to December 2019. The analysis was conducted using SPSS (Inc., Chicago, IL, USA), and descriptive statistics were employed to illustrate the findings.

**Results:**

We have found 14.4% gram-positive bacteria (n = 100) and 85.6% gram-negative bacteria (n = 595) among the 695 samples by gram staining methods. The most prevalent gram-positive and gram-negative bacteria present in wound infections were Staphylococcus spp. (81.5%) and Pseudomonas spp. (89%), respectively. Antimicrobials that were mostly resistant to gram-negative isolates were Amoxicillin (75.8%), Cefixime (75.5%), Cefuroxime (70.3%), and Ceftazidime (69.6%). On the other hand, cefixime and ceftazidime accounted for 73% of the resistance against gram-positive isolates, followed by amoxicillin (71%), and penicillin-G (69%). Meropenem was found to be the most sensitive antibiotic for gram-negative bacteria. Meropenem and Gentamycin were found to have a percentage of sensitivity for gram-positive bacteria. Based on the assessment of 13 different antimicrobial classes, the percentage of multi-drug resistant bacteria identified in gram-negative bacteria was 84% and in gram-positive bacteria was 79%. Among gram-negative bacterial isolates, 82% pseudomonas spp, 88.5% Klebsiella spp, and 91.6% Proteus spp were reported as multi-drug resistant. On the other hand, Pseudomonas spp, Klebsiella spp, and Proteus spp. were found to be multi-drug resistant in 82%, 88.5%, and 91.6% of gram-negative bacterial isolates, respectively. It was shown that staphylococcus aureus (81%) and staphylococcus spp (78.6%) became gram-positive among gram-positive isolates.

**Conclusion:**

According to this study, frequently isolated bacteria have a high frequency of MDR, which is the most pressing issue in public health. This study helps to manage the evidence-based treatment strategy and the urgency of early identification of drug-resistant bacteria that can reduce disease burden.

## Highlights

• Antimicrobial resistance has become one of the most pressing public health issues.
• Several multi-drug resistant bacteria were isolated from the wound infections and also demonstrated their antibiotic susceptibility.
• Antimicrobials that were mostly resistant to gram-negative isolates were Amoxicillin (75.8%), Cefixime (75.5%), Cefuroxime (70.3%), and Ceftazidime (69.6%).
• Cefixime and ceftazidime accounted for 73% of the resistance against gram-positive isolates, followed by amoxicillin (71%), and penicillin-G (69%).
• Meropenem was found to be the most sensitive antibiotic for both Gram positive and Gram negative bacteria.

## 1 Introduction

Antimicrobial resistance (AMR) has emerged as one of the most concerning issues in terms of mortality and economic burden [1]. Managing this issue effectively has proven challenging, especially in developing nations like Bangladesh, owing to the lack of relevant scientific findings, the lack of data sharing, the low health standards, and the low quality of drugs [2,3]. Moreover, self-medication by patients, unnecessary antibiotic prescriptions made by physicians without doing proper susceptibility testing on the bacteria, and the rapid and uncontrolled use of antimicrobials in agriculture and farming have all exacerbated the problem [4,5]. Wounds typically form when the skin epithelium and skin integrity deteriorate. Wounds generally form when the skin epithelium and skin integrity deteriorate. Thus, exposure to the subcutaneous tissues of wounds allows easy access to polymicrobes like bacteria, viruses, and fungi, and offers a nourishing and sustaining environment for the growth and multiplication of these organisms [6,7]. The environment of wounds is wet, warm, and nutritious, which promotes their colonization and proliferation and makes them more contagious [8]. Therefore, microbial invasion of the wound site by an imbalanced host immune response might eventually lead to chronic wound infection [9]. This chronic infection triggers longer hospital stays, which raises the patients' cost-effectiveness. Long-term, indiscriminate use of antibiotics causes major genetic changes in bacteria, which reduces the effectiveness of many different types of antibiotics and leads to the development of AMR [10]. As a result, the management of wound infections has become a serious issue due to the alarming rise in infections caused by the emergence of antibiotic-resistant bacteria [11].

According to several studies, the most common bacteria that cause wound infection are Staphylococcus aureus, Pseudomonas aeruginosa, E. coli, Acinetobacter spp, and Klebsiella spp [[12], [13], [14]]. Multi-drug resistant (MDR) microbes may survive for long periods of time and may multiply in the presence of minimal nutrients and have the capability of colonizing injured skin, which is a significant threat to public health globally. The epidemiological rate has a significant impact on the resistance pattern of wound-associated bacteria, which varies globally and in regional settings [9]. Studies conducted in many developing nations, including Africa, Ethiopia, Nigeria, and Ghana, have shown that the presence of AMR and MDR reduces the effectiveness of treatments for common wound infections [15,16]. Thus, the development of AMR and MDR leads to therapeutic failure, prolonged hospitalization, increased treatment cost, mortality, and the spread of MDR pathogens [17].

In this study, we investigated the causative agent of wound infection and assessed its AMR and MDR patterns. This study also aimed to examine the current situations in Bangladesh in order to better advise clinicians and microbiologists on how to manage infected wounds and to make them aware of the actual situations that they are now dealing with.

## 2 Materials and methods

### 2.1 Study areas and time frame

We have collected a total of 699 samples from the Lab Zone diagnostic center, Tangail, Bangladesh, over the period of 1 year (January 2018 to December 2019).

### 2.2 Ethical clearance

The specimens were collected in compliance with international safety rules and ethical standards, and the study was approved by the Institutional Ethics Review Committee of the Department of Biochemistry and Molecular Biology, Mawlana Bhashani Science and Technology University, Santosh, Tangail-1902, Bangladesh. Our study was conducted in accordance with the Declaration of Helsinki. This research is fully compliant with the STROCSS 2021 criteria [18]. The study approved by the ethical review committee of the Department of Biochemistry and Molecular Biology, Mawlana Bhashani Science and Technology University, Santosh, Tangail-1902, Bangladesh, with the certificate number MBSTU/BMB/TEST/6/2022/153.

### 2.3 Data collection

A total of 699 swab samples were collected from patients with various wound infections, including post-operative surgical wounds, burn wounds, and superficial and soft tissue infections. The age and sex of patients, the bacteria isolated, and the drug susceptibility profiles were retrieved from microbiology laboratory unit registration records using a standard data collection form. Laboratory records that had incomplete information on either age, sex, or culture and drug susceptibility test results were excluded from the analysis.

**Fig. 1:**
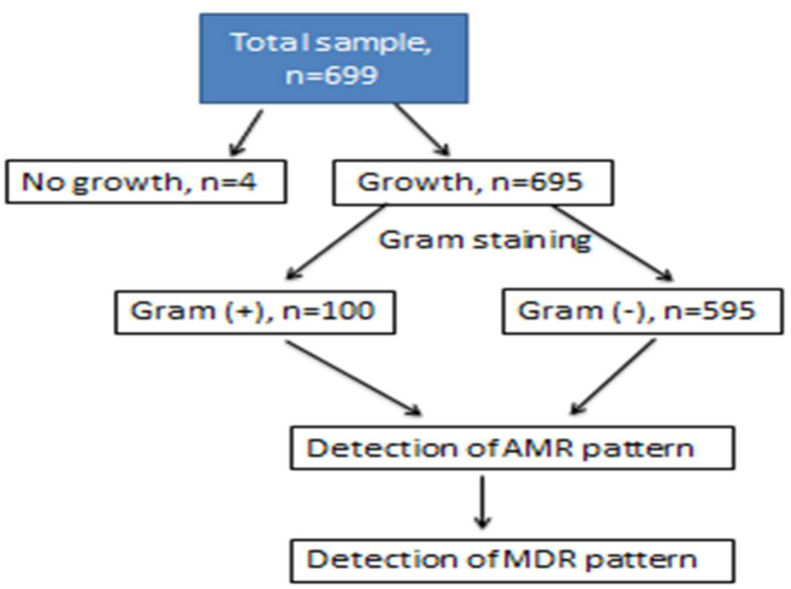
Workflow outline.

According to clinical laboratory guidelines, swab specimens were inoculated into various types of agar media such as Blood agar, MacConkey agar, Nutrient agar, and Potato Dextrose agar plates. The preliminary identification of the isolated bacteria was done based on colony form, size, shape, pigmentation, margin, and elevation. Different biochemical tests and Gram staining methods were employed to identify the isolated organisms. Then antibiotic susceptibility testing was performed. The culture plates were examined for microbial growth after proper incubation (at 37 °C overnight), and each plate was carefully observed. Finally, biochemical assays were performed in sterile media for the identification of bacterial isolates (Fig. 1).

### 2.4 Antimicrobial susceptibility pattern analysis

A standard disk diffusion technique reported by the Clinical and Laboratory Standards Institute (CLSI) using the Kirby-Bauer disk diffusion test on Mueller-Hinton agar was used to perform an antibiotic susceptibility test [19,20]. About 13 classes of antibiotics such as Aminoglycosides (Amikacin, Gentamicin), Tetracycline (Tetracycline), Carbapenems (Meropenem), Cephalosporin (Ceftriaxone, Ceftazidime, Cefixime, Cephalexin, Cephradine, Cefuroxime), Fluoroquinolone (Levofloxacin, Ciprofloxacin), Lincosamide (Clindamycin), Oxazolidinone (Linezolid), Penicillin (Penicillin-G, Amoxicillin), Sulfonamides (Sulfamethoxazole/Trimethoprim), Macrolides (Azithromycin), Nitrofuran (Nitrofurantoin) antibiotics, Azole antifungals (Fluconazole), polymyxin (Colistin) were involved in the testing. In this study, bacterial isolates from wound cultures were tested for AMR and MDR.

### 2.5 Statistical analysis

The analysis was conducted using IBM SPSS (Inc., Chicago, IL, USA) and Microsoft Excel. Frequency distribution, Cross-tabulation, and Bar charts were applied for the statistical estimation of the variables. The patterns of AMR and MDR were determined using descriptive statistics.

## 3 Results

### 3.1 Distribution of wound infections

**Fig. 2:**
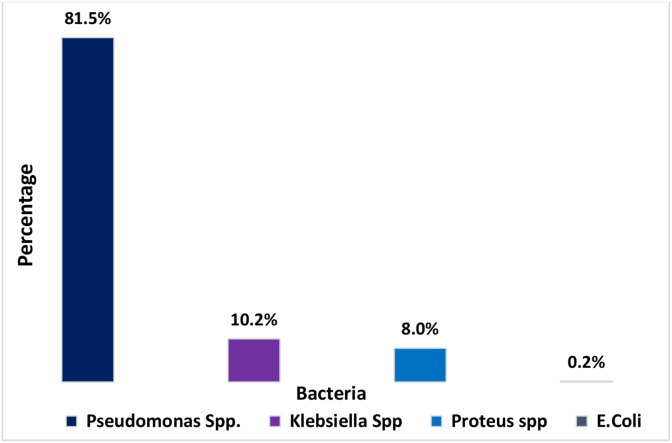
Distribution of the wound infection by the gram-negative bacteria.

**Fig. 3:**
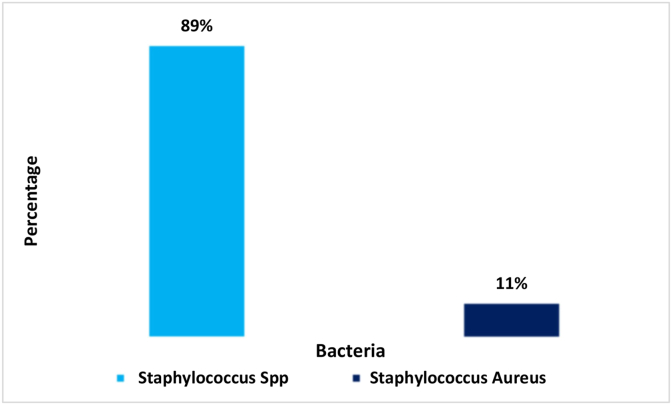
Distribution of the wound infection by the gram-positive bacteria.

In this study, we screened scripts from microbiological culture results for a variety of wound infection samples and analyzed their sensitivity reports. We found 6 types of bacteria from 669 isolates of wound samples, where 14.3% were gram-positive bacteria (n = 100), 99.4% were gram-negative bacteria (n = 595), and 0.6% were no growth (n = 4) (Table 1). Among gram-negative samples, 4 types of bacteria were detected: Pseudomonas Spp (n = 485) 81.5%, Klebsiella Spp (n = 61) 10.2%, Proteus Spp (n = 48) 8%, and *E. coli* (n = 1) 0.1% (Fig. 1). Gram-positive isolates contained only two types of bacteria; Staphylococcus Spp (n = 89) 89% and Staphylococcus Aureus (n = 11) (11%). (Fig. 3). Pseudomonas spp. was the most predominant among the gram-negative bacteria and Staphylococcus spp. for gram-positive bacteria (Fig. 2, Fig. 3).

**Table 1:** Gram staining result of bacterial isolates collected from various wound infection.

Gram staining	Sub types	Frequency	Percentage (%)
Total samples, n = 695	Gram (+)	100	14.4
No growth identified, n = 4	Gram (−)	595	85.6

### 3.2 Antibiotic resistance pattern of gram-negative bacteria

Overall, 22 antimicrobials of 13 types (Aminoglycosides, Tetracycline, Carbapenems, Cephalosporin, Fluoroquinolone, Lincosamide, Oxazolidinone, Penicillin, Sulfonamides, Macrolides, Nitrofuran antibiotics, Azole antifungals, polymyxin) were tested against gram-positive and gram-negative bacteria. Antimicrobial resistance to gram-negative isolates was found to be highest with amoxicillin (75.6%), cefixime (75.5%), cefuroxime (70.3%), and ceftazidime (69.6%). Pseudomonas spp was the most resistant to (Cefixime) CFM (74.6%), while Klebsiella spp and Proteus spp. were the most resistant to CN (Cephalexin) 90.1%, and CFM (Cefixime) 77%. All four types of gram-negative isolates were sensitive to Meropenem (Carbapenem group) (1.7% resistant) (Table 2).

**Table 2:** AMR pattern of Gram (−) bacteria cultured from wound infection.

Antibiotics resistance	Pseudomonas Spp. (n = 485)	Klebsiella Spp (n = 61)	Proteus Spp. (n = 48)	E.Coli (n = 1)	Total (n = 595)
AMI(%)	23 (4.7)	6 (9.8)	4 (8.3)	1 (100)	34 (5.7)
CTR(%)	194 (40)	34 (55.7)	25 (52)	0	253 (42.5)
TCY(%)	108 (22.3)	28 (45.9)	15 (31.2)	1 (100)	152 (25.5)
CN(%)	234 (48.3)	55 (90.1)	33 (68.7)	1 (100)	323 (54.3)
LEV(%)	53 (10.9)	14 (22.9)	6 (12.5)	0	73 (12.3)
CFM (%)	362 (74.6)	49 (80.3)	37 (77)	1 (100)	449 (75.5)
CIP(%)	118 (24.3)	23 (23)	14 (29.2)	0	155 (26)
CLN(%)	217 (44.7)	52 (85.2)	32 (66.7)	1 (100)	302 (50.8)
LIN (%)	187 (38.5)	49 (80.3)	23 (47.9)	1 (100)	260 (43.7)
PG(%)	280 (57.7)	28 (45.9)	33 (68.7)	1 (100)	342 (57.5)
COL(%)	286 (58.9)	25 (40.9)	25 (52)	1 (100)	346 (58.2)
MER(%)	8 (1.6)	2 (3.3)	0	0	10 (1.7)
CED(%)	226 (46.6)	51 (83.6)	29 (60.4)	1 (100)	307 (51.6)
AZI(%)	169 (34.8)	29 (47.5)	24 (50)	1 (100)	223 (37.8)
AMC(%)	360 (74.2)	52 (85.2)	38 (79.1)	1 (100)	451 (75.8)
TMP(%)	234 (48.2)	35 (57.4)	19 (39.6)	1 (100)	289 (48.6)
GEN(%)	40 (8.2)	9 (14.7)	5 (10.4)	0	54 (9)
NIT (%)	188 (38.7)	26 (42.6)	20 (41.6)	1 (100)	235 (39.5)
CAZ (%)	333 (68.8)	42 (68.8)	1 (2)	1 (100)	414 (69.6)
CXM(%)	332 (68.4)	48 (78.7)	1 (2)	1 (100)	418 (70.3)
FCZ (%)	209 (43)	27 (44.3)	20 (41.6)	1 (100)	257 (43.2)
COT (%)	195 (40.2)	8 (13.1)	28 (58.3)	1 (100)	233 (39.2)

### 3.3 Antibiotic resistance pattern of gram-positive bacteria

Among 22 different antimicrobials, CFM (cefixime) and CAZ (Ceftazidime) were (73%) the most highly resistant antimicrobials against gram-positive isolates. Amoxicillin and penicillin-G (penicillin group) were 71% and 69% resistant, respectively. The resistance to cefixime, ceftazidime, penicillin-G, and amoxicillin was observed in 71.9%, 70.8%, 68.5%, and 68.5%, respectively, in Staphylococcus Spp. The rates of resistance of Staphylococcus Aureus to Cefixime, Amoxicillin, Penicillin-G, and Ceftazidime were 81.8%, 90.9%, 72.7%, and 90.9%, respectively. No Meropenem-resistant or Gentamycin-resistant *Staphylococcus* aureus was detected, nor were there any Meropenem-resistant Staphylococcus spp (Table 3).

**Table 3:** Antibiotic resistance pattern of Gram-positive bacteria from wound infection.

Antimicrobials	Staphylococcus Aureus (n = 11)	staphylococcus spp (n = 89)	Total (n = 100)
AMI(%)	1 (9.0)	1 (1.1)	2 (2)
CTR (%)	3 (27.3)	28 (31.5)	31 (31)
TCY(%)	2 (18.2)	22 (24.7)	24 (24)
CN(%)	7 (63.6)	45 (50.6)	52 (52)
LEV (%)	2 (18.2)	7 (7.9)	9 (9)
CFM(%)	9 (81.8)	64 (71.9)	73 (73)
CIP(%)	4 (36.4)	20 (22.5)	24 (24)
CLN (%)	7 (63.6)	44 (49.4)	51 (51)
LIN(%)	5 (45.5)	31 (34.8)	36 (36)
PG (%)	8 (72.7)	61 (68.5)	69 (69)
COL (%)	7 (63.6)	48 (53.9)	55 (55)
MER (%)	0	0	0
CED (%)	5 (45.5)	46 (51.6)	51 (51)
AZI (%)	9 (81.8)	27 (30.3)	36 (36)
AMC(%)	10 (90.9)	61 (68.5)	71 (71)
TMP(%)	6 (54.5)	42 (47.2)	48 (48)
GEN	0	4 (4.5)	4 (4)
NIT (%)	2 (18.2)	42 (47.2)	44 (44)
CAZ (%)	10 (90.9)	63 (70.8)	73 (73)
CXM(%)	6 (54.5)	51 (57.3)	57 (57)
FCZ (%)	5 (45.5)	42 (47.2)	47 (47)
COT (%)	3 (27.3)	53 (59.5)	56 (56)

### 3.4 Multi-drug resistance pattern of gram-positive and gram-negative bacteria

MDR was previously defined as resistance to three or more antibiotic classes in gram-positive [[21], [22], [23]] and gram-negative [[24], [25], [26]] bacteria. It was observed that 499 (84%) gram-negative bacterial isolates were MDR, whereas 11.3% were sensitive to all 13 types of antibiotic classes. Among gram-negative bacterial isolates, 82% (401) of pseudomonas spp, 88.5% (54) Klebsiella spp, and 91.6% (44) Proteus spp were determined as MDR. 13 types of antimicrobials were shown to be effective against 12.9% pseudomonas spp, 3.3% Klebsiella spp, and 4.2% Proteus spp. On the contrary, 17% of gram-positive (17.9% staphylococcus spp and 9% staphylococcus aureus) were effective against 13 types of antimicrobial classes and 79% of gram-positive isolates were evaluated as MDR. Then, 78.6% of staphylococcus spp. and 81% of staphylococcus aureus were observed as MDR. Staphylococcus aureus was found to be the most resistant bacteria, with 45.5% resistant to 11 different antimicrobials (Table 4).

**Table 4:** MDR pattern of Gram-negative bacteria isolated from infected wounds.

Bacteria	R0 (%)	R1-R3 (%)	R4 (%)	R5 (%)	R6 (%)	R7 (%)	R8 (%)	R9 (%)	R10-R13 (%)	MDR (%)	Antimicrobial class used to define MDR
Pseudomonas Spp. n = 485 (%)	63 (12.9)	13 (2.7)	53 (10.9)	81 (16.7)	82 (16.9)	87 (17.9)	64 (13.2)	26 (5.7)	8 (1.7)	401 (82)	Aminoglycosides, Tetracycline, Carbapenems, Cephalosporin, Fluoroquinolone, Lincosamide, Oxazolidinone, Penicillin, Sulfonamides, Macrolides, Nitrofuran antibiotics, Azole antifungals, polymyxin.
Klebsiella Spp. (n = 61)	2 (3.3)	2 (3.3)	3 (4.9)	6 (9.8)	10 (16.3)	13 (21.3)	8 (13.1)	9 (14.7)	5 (8.2)	54 (88.5)	
Proteus Spp. (n = 48)	2 (4.2)	1 (2)	3 (6.3)	4 (8.3)	11 (22.9)	11 (22.9)	7 (14.6)	5 (10.4)	3 (6.2)	44 (91.6)	
Total n = 594	67 (11.3)									n = 499 (84)	
Staphylococcus Spp. n = 89	16 (17).	0	0	14 (15.7)	11 (12.3)	22 (24.7)	17 (19.1)	5 (5.6)	1 (1.1)	70 (78.6)	
Staphylococcus Aureus. (n = 11)	1 (9)	1 (9)	0	2 (18.1)	5 (45.5)	1 (9)	0	1 (9)	0	9 (81)	
Total n = 100	17%									(n = 79) 79%	

R0: Sensitive against all selected antimicrobials classes; R1-R3: Resistant to one to three antimicrobials classes; R4: Resistant to four antibiotic classes; R5: Resistant to five antibiotic classes; R6: Resistant to six antibiotic classes; R7: Resistant to seven antibiotic classes; R8: Resistant to eight antibiotic classes; R9: Resistant to nine antibiotic classes; R10-R11: Resistant to ten to thirteen antibiotic classes; MDR: Resistant to more than 3 antimicrobial.

## 4 Discussion

Despite advancements in surgical methods and the use of antibiotic prophylaxis, wound infections continue to be a major public health issue. Infection control after surgery is still a major concern for doctors all over the world due to AMR [27]. Antibiotic misuse to combat bacterial infections leads to an increase in bacterial resistance. The list of microorganisms that are commonly associated with wound infection has been illustrated in our study, and the prevalence of MDR bacteria in wound infection was also identified. We concluded that Pseudomonas spp. followed by Staphylococcus spp, Klebsiella spp, and Proteus spp. were the most prevalent isolates associated with wound infection. This is also supported by other studies [5,11,28,29]. More than three-thirds of gram-negative bacteria were Pseudomonas spp. and nearly 90% of gram-positive bacteria were Staphylococcus spp. in this study.

In our investigation, Meropenem (Carbapenem group), followed by Amikacin and Gentamycin (Aminoglycoside group), were the most effective antibiotics against gram-negative microbes. A similar result was reported in a previous study published in 2013 by Lucinda J Bessa et al. and in 2017 by Víctor Silva et al. [30,31]. Proteus species and E. coli were not found to be resistant to Meropenem, whereas Pseudomonas spp. and Klebsiella spp were resistant to Meropenem at 1.6% and 3.3%, respectively. Resistance to amikacin and gentamycin in gram-negative microbes ranges from 5.7% to 9%. Approximately three-thirds of Pseudomonas Spp were resistant to Cefixime, Ceftazidime, and Amoxicillin. Klebsiella spp. was shown to be the most resistant gram-negative isolate. On average, 80%–90% of these isolates were resistant to Cefalexin, Cefixime, Clindamycin, Linezolid, Cefradine, Amoxicillin, and Cefuroxime.

According to our analysis, the most effective antimicrobials against gram-positive bacteria were Meropenem, Gentamycin, and Amikacin. We did not find Meropenem-resistant Staphylococcus spp. and Staphylococcus Aureus. Cefixime, Ceftazidime, Amoxicillin, and Penicillin-G were identified as the most resistant antimicrobials to gram-positive isolates, with over 70% resistance. More than 90% of staphylococcus aureus were reported to be resistant to Amoxicillin and ceftazidime [32] and 81.8% of staphylococcus aureus were found to be resistant to azithromycin and cefuroxime [32]. More than 50% of all Staphylococcus spp. were resistant to Levofloxacin (50.6%), Cefixime (71.9%), colistin (53.9%), Penicillin-G (68.5%), Cefradine (51.6%), Amoxicillin (68.5%), Ceftazidime (70.8%), and Trimethoprim/sulfamethoxazole (59.5%).

In comparison to gram-positive bacteria (79%), gram-negative (84%) bacteria isolated from wound infections had a somewhat greater percentage of MDR. Among gram-negative bacterial isolates, Proteus spp showed the highest rate of MDR (91.6%). The MDR rates of Klebsiella spp (88%) and Pseudomonas spp. (84%) were slightly lower than those of Proteus spp. Pseudomonas spp (1.2%), Klebsiella spp (8.2%), and Proteus spp (6.2%) showed resistance to 10–13 types of antimicrobials. In our investigation, the total MDR rate in the case of gram-negative bacteria was higher than previously reported [11]. Gram-positive bacteria such as Staphylococcus spp. (78.6%) and Staphylococcus aureus (81%) had higher MDR percentages. Although no strains of Staphylococcus aureus were found to be resistant to more than nine antimicrobials, only 1.1% of Staphylococcus spp. were.

## 5 Conclusion

The most common isolates discovered in our analysis were Pseudomonas spp, Staphylococcus spp, Staphylococcus aureus, Klebsiella spp, Proteus spp, and E. coli. The most sensitive antibiotics against gram-positive and gram-negative microbes were Meropenem (Carbapenem group), Amikacin, and Gentamycin (Aminoglycoside group). Most commercially available antibiotics used in Bangladesh revealed significant levels of resistance amongst isolates. This research found a high prevalence of MDR among frequently isolated pathogens. The continual emergence of MDR microbes is an alarming and very serious issue. Wound infection can be prevented with the early detection of drug-resistant microbes and an evidence-based treatment plan.

## Ethical approval

This study was approved by ethical review committee of the Department of Biochemistry and Molecular Biology, Mawlana Bhasani Science and Technology University and Lab Zone diagnostic center, Tangail, Bangladesh, with the certificate number MBSTU/BMB/TEST/6/2022/153.

## Sources of funding

No funding from the sponsors.

## Author contribution

MJI conceptualized this study. FAN, GB, and SI drafted the manuscript. MJI, AI, and GB critically reviewed the manuscript. SA, RAJ, and TCS collected the information and data analysis.

## Trial registry number

1. Name of the registry: NA.

2. Unique Identifying number or registration ID: NA.

3. Hyperlink to your specific registration (must be publicly accessible and will be checked): NA.

## Guarantor

Mohammod Johirul Islam, Associate Professor, Dept. of Biochemistry and Molecular Biology, MBSTU.

## Consent

During the entry of the patients in our study, patients were fully informed.

## Provenance and peer review

Not commissioned, externally peer reviewed.

## Declaration of competing interest

No conflict of interest from the authors.
